# Visual rating versus volumetric segmentation of white matter hyperintensities for cognitive assessment: a memory clinic-based comparison and systematic review

**DOI:** 10.1016/j.cccb.2026.100564

**Published:** 2026-10-03

**Authors:** Geke G.C.E.A. van Dijk, Camiel V.J. Box, Farog Faghir, Joy Martens, Janne M. Papma, Francesco U.S. Mattace Raso, Esther Van Den Berg, Meike W. Vernooij, Frank J. Wolters

**Affiliations:** a Dept. of Epidemiology, Erasmus MC University Medical Center, Rotterdam, the Netherlands; b Dept. of Radiology & Nuclear Medicine and Alzheimer Center Erasmus MC, Erasmus MC University Medical Center, Rotterdam, the Netherlands; c Dept. of Neurology and Alzheimer Center Erasmus MC, Erasmus MC University Medical Center, Rotterdam, the Netherlands; d Dept. of Geriatrics and Alzheimer Center Erasmus MC, Erasmus MC University Medical Center, Rotterdam, the Netherlands

**Keywords:** White matter hyperintensities, Cognition, Automated segmentation, Visual rating, Fazekas

## Abstract

**Introduction:**

Clinical impression of WMH commonly relies on visual rating scales (VRS), yet may improve through volumetric segmentation. Comparative studies, particularly considering clinical outcomes, are scarce.

**Methods:**

We conducted a retrospective, cross-sectional study among 246 memory clinic patients. We compared WMH assessed through VRS (Fazekas) to automated volumetric segmentation (Quantib), through correlation and explained variance in neuropsychological assessment across six cognitive domains, above and beyond age, sex, and educational attainment in linear regression models. Results were contextualised through systematic literature review until 30 September 2022.

**Results:**

Fazekas scores and WMH volumes were strongly correlated in the memory clinic population (ρ=0.83, p < 0.001), with little overlap in volumes between Fazekas categories. This was supported by similar correlations observed in 24 studies included in the systematic review and 13 in meta-analysis. Jointly with demographics, VRS and segmentation explained 6–19% of variance in cognitive domains in the memory clinic population. Volumetrics explained at most 2% more variance than visual rating, most substantial in MMSE (2%), in contrast to executive function (-1%). The systematic review identified three other comparative studies on cognitive outcome, each reporting similar correlations with volumetrics and visual rating scales. Previously proposed cut-offs in WMH volume that approximate Fazekas categories (3.4, 9.6 and 17.1 mL) showed classification accuracies of 59%, 70% and 78% in our cohort, with optimal thresholds in our sample at 1.6, 6.0 and 19.8 mL.

**Discussion:**

VRS and volumetric segmentation of WMH are highly correlated. Small improvements in explained variance in cognition with volumetrics were inconsistent across cognitive domains

## Highlights

• Visual rating and automated volumetric measures of WMH are highly correlated.
• Volumetric WMH measures add only limited explanatory value beyond visual ratings, with no gain for executive function.
• Fazekas categories mostly correspond to distinct WMH volumes, enabling translation between both measures for data harmonisation.

## Background

Cerebrovascular changes are highly prevalent with aging, and 84% of individuals with dementia exhibit vascular pathology alongside Alzheimer’s disease pathology [1]. As targeted disease-modifying treatments are emerging, precise diagnosis of the relative contribution of vascular and neurodegenerative processes in the individual patient becomes increasingly important [2]. This requires reliable and fine-grained assessment of WMH levels. White matter hyperintensities (WMH) are a key imaging biomarker of cerebral small vessel disease [3], associated with poorer cognitive performance and increased risk of dementia [4]. For the assessment of WMH in patients presenting with cognitive complaints in routine clinical practice, WMH are typically assessed by radiologists using semi-quantitative visual rating scales (VRS) like the Fazekas and Scheltens scales [5]. These VRS provide coarse, categorical estimates of WMH [3,6]. Although widely applied, VRS are observer-dependent, require training, and are not very sensitive to subtle changes, resulting in one third of radiologists reporting limited confidence in their application of VRS for scan interpretation [7]. In research settings, WMH are nowadays regularly quantified using volumetric segmentation. Advances in automated segmentation have made this process faster and more reproducible than manual delineations [8]. Volumetric methods appear promising, as they provide observer-independent, continuous measurement, and greater sensitivity to subtle changes or spatially specific lesion patterns [3,7]. Yet, volumetric WMH assessment has not been integrated into routine diagnostic workflow, in part due to logistical barriers, but foremost because of a lack of direct comparative evidence on how volumetric and visual WMH assessment relate to clinically relevant outcomes, including cognitive performance [7].

We aimed to determine the correlation between visual rating and volumetric segmentation of WMH and compare their clinical utility for the assessment of WMH as a contributor to cognitive impairment among patients attending a memory clinic. To evaluate consistency across rating scales, applied tools, cognitive domains and populations, we contextualise results through systematic review and meta-analysis of published literature.

## Methods

### Study population

In this cross-sectional study, we included patients who visited the Alzheimer Centre of the Erasmus MC University Medical Centre, a tertiary referral centre in Rotterdam, the Netherlands. As part of the routine diagnostic work-up at the memory clinic, patients underwent a standardised interview on medical history, physical and neurological examination, neuropsychological assessment, brain MRI and, when indicated, additional diagnostic procedures, followed by a final clinical diagnosis. Patients were invited to participate in scientific research by providing consent for the use of their clinical data for research purposes. Of 1507 patients who visited the outpatient clinic between 2016 and 2021, 869 (58%) underwent brain MRI either locally or in the referring centre. Of those, 288 had a scan that included both high-resolution T1 images and a FLAIR sequence to allow volumetric segmentation of WMH with Quantib. A detailed neuropsychological assessment dating <1 year from the MRI was done in 253 of 288 patients. A further 7 patients were excluded because of segmentation errors (e.g., due to prior ischemia), leaving 246 patients for analysis in the current study.

### Brain MRI protocol and segmentation of white matter hyperintensities (WMH)

All patients underwent scanning on a 1.5T or 3.0T scanner (GE Medical Systems, Philips Medical Systems, or Siemens Healthineers), including at least Fluid Attenuated Inversion Recovery (FLAIR) sequences. Volumetric segmentation of WMH was done using Quantib (DeepHealth, Somerville, MA, USA), a software tool for automated WMH segmentation (https://deephealth.com/quantib/). Outlier segmentations were visually inspected for accuracy if WMH volumes deviated from the Fazekas class-specific median >1.5 times the interquartile range.

### Visual rating of WMH

Visual rating of WMH in accordance with the Fazekas scale was performed by consultant neuroradiologists as part of routine clinical diagnostic work-up, blinded to volumetric WMH assessment [6]. In order to use readings that were most reflective of use of the Fazekas scale in clinical practice, we did not perform research-specific readings for the current study. Fazekas scores were extracted from the neuroradiology reports using a standardised form. If the Fazekas score was not reported but the report explicitly mentioned the absence of WMH, it was assigned a Fazekas score of 0. For 15 patients without sufficient information on WMH in their neuroradiology reports, scans were additionally rated by a consultant neuroradiologist (M.W.V.). Whenever reports indicated a range of scores (i.e., 0 to 1, 1 to 2, or 2 to 3), the higher score was used.

### Cognitive performance

Patients underwent neuropsychological testing as part of routine diagnostic work-up, covering a range of cognitive domains including memory, working memory, language, information processing speed, and executive functioning. While multiple tests were administered to most patients by default, heterogeneity in clinical presentation warranted variation in the specific tests for any individual patient. We prespecified a set of tests that provided good coverage of domains, with the Mini-Mental State Examination (MMSE) for global cognition [9]. For memory, we used scores on the Visual Association Test (VAT) and the delayed recall of the Rey Auditory Verbal Learning Test (RAVLT) [10,11]. Working memory was assessed by the Digit Span task of the Wechsler Adult Intelligence Scale (WAIS-IV) [12]. Language was derived from the Boston Naming Test and two verbal fluency tasks (i.e., animal and category fluency) [13,14]. Information processing speed was based on the Trail Making Test part A (TMT-A) and the first and second trial of the Stroop test [15,16]. The Stroop trial 3 and TMT-B were used to assess executive functioning, accounting for information processing performance by subtracting the mean of Stroop trials 1 and 2 (for Stroop 3) or TMT-A (for TMT-B), and then dividing by the duration of the processing speed trials. Aborted Stroop 3 or TMT-B tests were imputed on the 95th percentile score, assuming tests were uncompleted due to cognitive impairment.

### Systematic review

On 30 September 2022, we searched PubMed, Embase and the Cochrane Library for original research studies in the English language that performed a direct comparison of visual rating scales (VRS) and volumetric assessment of WMH on brain MRI of adult participants. The detailed search strategy is presented in the Supplementary Material S1. The review was inadvertently not pre-registered.

A flow chart of the search is presented in Fig. 1. All studies were first screened for eligibility based on title and abstract by two independent reviewers (G.C.E.A.D. and F.F.). Case reports, review articles, and animal studies were excluded. Subsequently, the same investigators independently evaluated the full-text of the remaining articles for eligibility. Discrepancies between reviewers were solved by consensus discussion. When multiple studies reported estimates of the same sample, we included the study reporting the largest sample size.

**Fig. 1:**
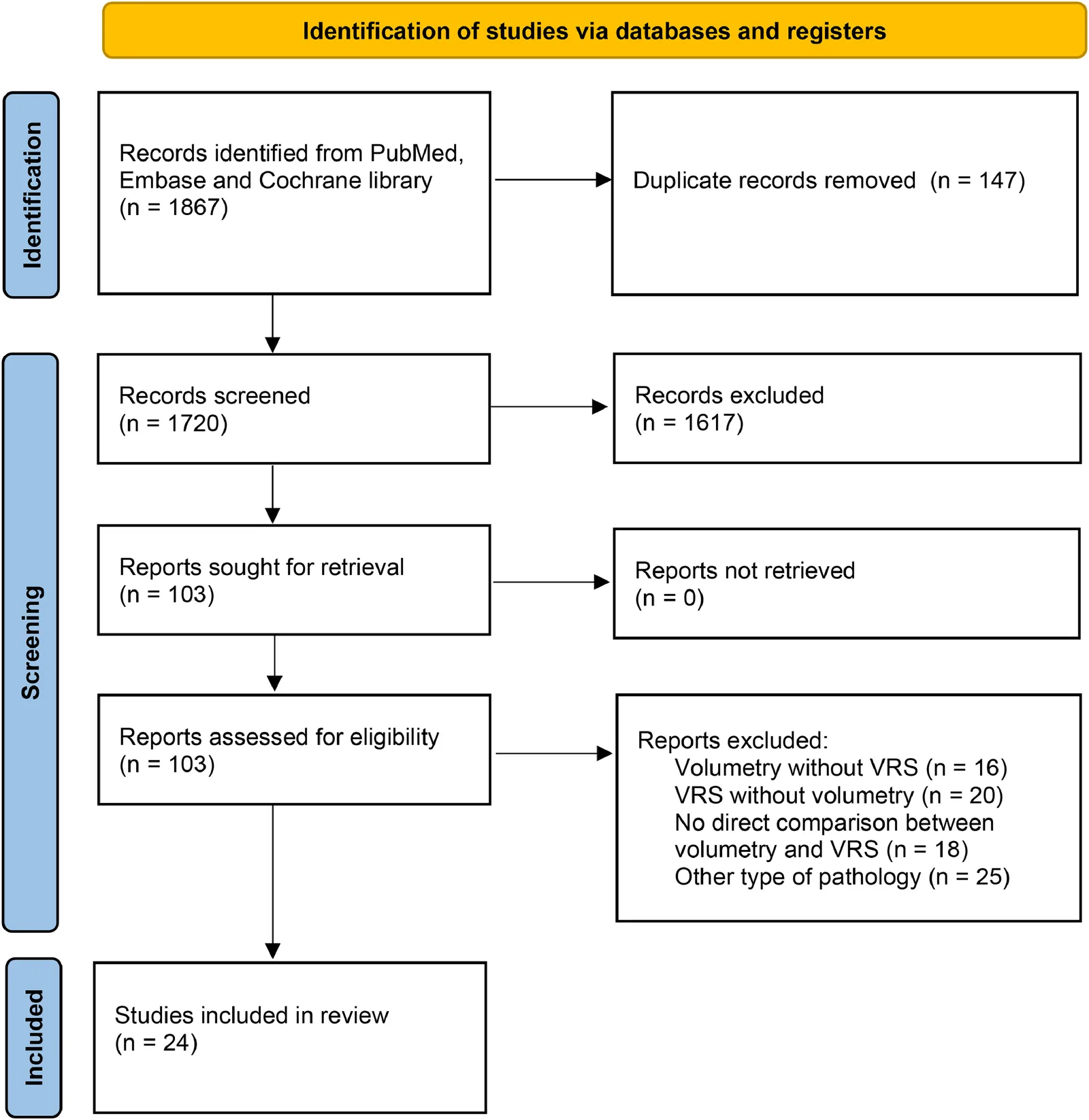
Systematic review flow diagram.

The two investigators (G.C.E.A.D. and F.F.) independently extracted the necessary data from the included articles, using a prespecified template. Extracted information included study characteristics (first author, year of publication, study design and setting), study population (country, sample size, age, follow-up time, sex, inclusion and exclusion criteria, diagnostic criteria), imaging protocol (field strength, sequences, image resolution, slice thickness, segmentation software), imaging assessment (applied VRS, number and experience of the rater(s), blinding, and inter- and intra-rater reliability), and outcome measures (e.g., correlation coefficients). Discrepancies were solved through consensus discussion with a third reviewer (F.J.W.). Study quality was evaluated based on the reporting of rater characteristics, blinding procedures, and inter-rater reliability, as well as clarity of the applied visual rating scale and analytic methods. These aspects were summarized descriptively (Supplementary material S2) and considered when interpreting results.

### Statistical analysis

Demographic data of the memory clinic study population were complete, but application of cognitive test results varied between patients, resulting in unavailable data for some patients on MMSE score (n = 71, 28.9%), working memory (n = 109, 44.3%), memory (n = 55, 22.4%), language (n = 19, 7.7%), processing speed (n = 57, 23.2%), and executive functioning (n = 61, 24.8%). We computed cognitive domain composite scores by averaging the standardized scores of the available tests within that domain. Domain scores were calculated if at least one of the domain’s tests were available. Results on time-based tests were inverted, such that higher scores indicate better performance on all tests. MMSE scores as well as domain scores for processing speed and executive functioning were transformed through rescaling to obtain a roughly normal distribution of the data [17]. TMT-A, TMT-B and Stroop-3 scores were transformed using the box-cox method with λ of −0.181, 0.101, and −0.303, respectively [18]. Summary statistics per test are shown in Supplementary Material S3.

In the memory clinic sample, we used the Spearman rank test to assess the correlation between the Fazekas score and WMH volumes, and visualised the distribution of WMH segmented volumes across Fazekas score groups in boxplots. We determined the variance in cognitive performance explained by the Fazekas score and by WMH volumes, expressed as the R^2^ from linear regression models that also included age, sex, and educational attainment (ordinal Verhage score), and compared these to a model with age, sex and education. We also compared the strength of association by comparing the standardised beta coefficients, along with their 84% confidence intervals [19], for Fazekas score and WMH volume from the linear regression models. Adding a quadratic term for WMH volume did not materially improve model fit. To allow comparison with continuous volumetric data, Fazekas score was entered into the model as an ordinal variable. We verified assumptions for linear regression through visual inspection of QQ-plots (normality of residuals) and residual plots (linearity and homoscedasticity), and the Durbin Watson test for independence of residuals. For processing speed and executive functioning, Q-Q plots showed slight deviations from the diagonal at the tails (Supplementary material S4), prompting sensitivity analyses for each component test.

In additional sensitivity analyses, we (i) included intracranial volume (ICV) in the WMH volume models, (ii) included Fazekas score as a categorical variable in the models compared to WMH volume in quartiles, (ii) repeated analyses using the non-imputed TMT-B and Stroop 3 scores, and (iv) repeated analyses among participants with Fazekas >0.

We used random-effects meta-analysis to pool our results with those found in the systematic review when at least three eligible studies using comparable methods were available. Studies reporting multiple correlation coefficients for different segmentation software were first internally meta-analysed (Supplementary material S5), after which the pooled outcome estimate was included in the overall meta-analysis. In case of repeated imaging, we used the sample size of unique participants for meta-analysis, including the first scan only. We constructed forest plots to visualise the effect sizes, and assessed heterogeneity using Higgins’ I^2^ [20].

Finally, from the studies identified in the systematic review that reported distributions of WMH across Fazekas categories, we extracted optimal cut-off points for determining Fazekas scores based on WMH volumes. Accuracy of these cut-offs was validated in the Alzheimer Centre Erasmus MC population. We also assessed optimal cut-off points within the data of our memory clinic population, using the Youden index.

All analyses were performed in R (v4.3.3). Meta-analysis was performed using the “metafor” package. Alpha was set at .05.

## Results

### Study population

Among a total of 246 included patients (mean age 67.5 years, 57.7% male), 62 (25.2%) had subjective cognitive complaints, 36 (14.6%) were diagnosed with mild cognitive impairment, and 91 (37.0%) with dementia. Dementia was most commonly attributed to Alzheimer’s disease (24.1%), frontotemporal dementia (17.2%), and vascular/mixed dementia (13.8%). Patient characteristics are presented in Table 1. Compared with all other 1261 patients who attended the outpatient clinic during the study period, included participants were slightly older (69.7 versus 67.5 years, *P* = 0.04), but did not differ in gender distribution (42.3 vs. 45.6% women, *P* = 0.30).

**Table 1:** Patient characteristics of the memory clinic population.

Characteristic	Value (N = 246)
Age, years	67.5 (±10.4)
Male sex	142 (57.7%)
Education	
Lower	126 (51.2%)
Middle	60 (24.4%)
Higher	58 (23.6%)
Clinical diagnosis	
Subjective cognitive complaints	62 (25.2%)
Mild cognitive impairment	36 (14.6%)
Dementia	91 (37.0%)
Other	26 (10.6%)
Postponed diagnosis	31 (12.6%)
MMSE score	24.2 (±4.9)
WMH volume, mL (median, IQR)	4.1 (1.1–11.3)
Fazekas	
Score 0	53 (22%)
Score 1	97 (39%)
Score 2	65 (26%)
Score 3	31 (13%)

NOTE. Numbers are presented as means±SD for continuous variables, and absolute counts (%) for categorical variables, unless specified otherwise. Educational attainment is shown in categories for descriptive purposes. Abbreviations: SD, standard deviation; MMSE, Mini Mental State Examination; WMH, white matter hyperintensity; mL, milliliter; IQR, interquartile range.

Correlation between Fazekas score and WMH volumetry was 0.83 (*P* value <.001). Although the range and dispersion of volumes increased with higher Fazekas score, there was little overlap of WMH volumes between Fazekas categories (Fig. 2).

**Fig. 2:**
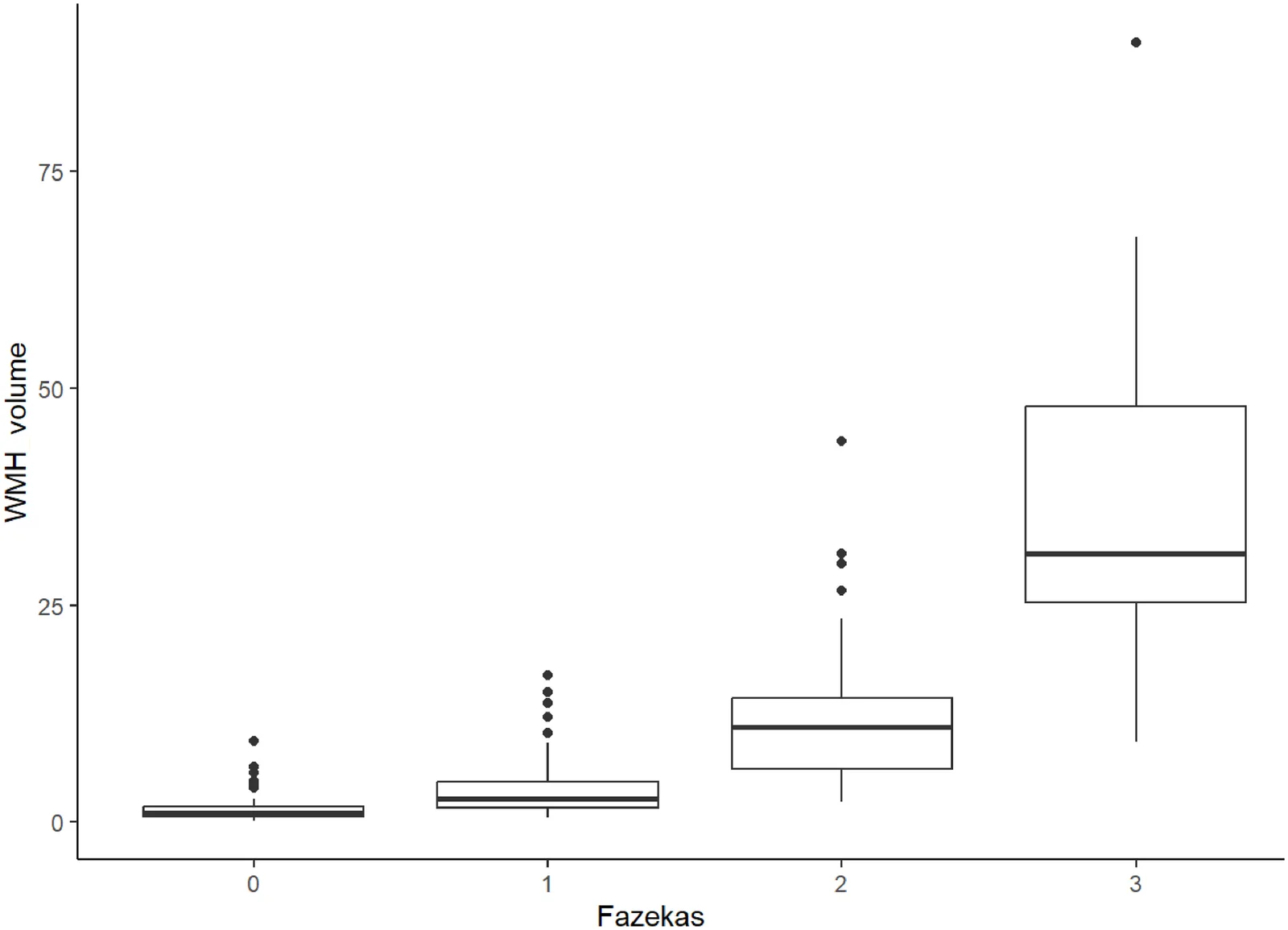
Correlation between volumetric segmentations and Fazekas score.

**Subtitle:** Boxplots of WMH volumes per Fazekas score in the memory clinic population (N = 246). NOTE. Boxes represent interquartile range (IQR) with median marked by the middle line. Whiskers represent 1.5 IQR distance from the first and third quartiles, with outliers in WMH volume beyond these upper and lower bounds presented as individual dots.

When exploratively examining the associations between WMH levels and the different cognitive domains, the direction of effect was consistent with negative effects of WMH on performance on all but one of the cognitive domains (mean difference [MD] per domain ranging from –0.03 to –0.17; Table 2). Confidence intervals often included the null. Estimates were similar for the separate component tests of the domain scores (Supplementary material S6).

**Table 2:** Comparison of the Fazekas score and volumetric segmentation in relation to cognitive assessment.

Cognitive domain	Base model without WMH	Fazekas score		WMH volumetry segmentation	
	R^2^	Estimate (84% CI)	R^2^	Estimate (84% CI)	R^2^
Global (MMSE)^a^	0.06	−3.16 (−5.64; −0.68)	0.08	−4.09 (−6.26; −1.93)	0.10
Memory	0.15	−0.03 (−0.13; 0.08)	0.15	−0.07 (−0.17; 0.03)	0.15
Working memory	0.16	−0.05 (−0.18; 0.08)	0.16	−0.17 (−0.31; −0.02)	0.17
Language	0.17	−0.07 (−0.15; 0.02)	0.18	−0.13 (−0.21; −0.04)	0.19
Processing speed	0.10	0.01 (−0.09; 0.12)	0.10	−0.16 (−0.26; −0.05)	0.12
Executive functioning	0.05	−0.08 (−0.16; −0.01)	0.07	−0.08 (−0.15; −0.0003)	0.06

NOTE. Cognitive domains were included in the models as outcome, Fazekas score and WMH volumetry segmentation as continuous determinants. All models include age, sex and educational level. Estimates reflect the change in respectively rescaled MMSE score and cognitive domain composite score per unit increase in Fazekas score and 1 SD increase in WMH volume. The 84% confidence intervals (CI) facilitate comparison of any difference between the estimates for Fazekas score and WMH volumes.

a After transformation to normalise distribution.

The effect estimates for the associations with different cognitive domains were numerically somewhat larger for WMH volume than for Fazekas, but did not differ significantly between methods as the 84% CIs overlapped for all measures of cognition (Table 2). Across all cognitive domains, we found only small differences in explained variance between models including the Fazekas score and volumetric segmentations (Table 2). Explained variance differed no >1 to 2% between the two methods, generally in favour of volumetric segmentations. Compared to the models with only demographics, Fazekas score added 1 to 2% explained variance, whereas WMH volumetry added 1 to 4% explained variance in most domains. As such, WMH volumes marginally outperformed Fazekas scores with respect to explaining variance in global cognition (i.e., MMSE score) and to a lesser extent processing speed and language. In contrast, for executive functioning Fazekas score added more (2%) than WMH volume (1%), and neither method improved model fit for memory function (Table 2).

In sensitivity analyses, outcomes were similar when modelling WMH volume per quartile and Fazekas score as a categorical rather than ordinal variable (Supplementary material S7). Results were also similar for the different subtests that composed the domains scores of information processing and executive function (Supplementary material S6) and were unchanged in complete case analysis (data not shown). Adding quadratic terms for WMH volume or intracranial volume to the model did not change the results. Similar findings were observed in the subset of patients with Fazekas score >0.

### Systematic review: study characteristics

Of 1720 unique studies identified, 24 fulfilled the eligibility criteria and were included (Fig. 1). Study characteristics are presented in Table 3. Jointly, studies included 7578 participants (range: 20-1045), with a median age across studies of 70.8 years (range: 60.9 to 92.0 years). Roughly half of participants in the included studies were male (median 51.1%, range: 32.3% to 61.7%). Year of publication ranged from 2003 to 2022, with half of studies published since 2016. Of the included studies, 17 (71%) used automated segmentation methods, five (21%) used manual delineation, and two (8%) used a combination of both. The 19 studies using automated segmentation applied 22 different software tools, none of which was used across more than two different studies. The most used visual rating scale was the Fazekas scale (n = 15), followed by the Scheltens scale (n = 9), and the ARWMC (n = 6). 17 out of 24 studies (71%) reported a Spearman rank coefficient for the correlation between VRS and volumetric WMH (Fig. 3 and Supplementary material S8). Other analyses used were Kendall rank correlation, ANOVA, Kendall W test and linear regression.

**Table 3:** Study characteristics systematic review.

First author	Year	Data origin	Setting	Sample size	Mean age	Male %	Segmentation method	Volumetric software	Visual Rating Scale
Andere [21]	2022	Rhode Island Hospital	Stroke clinic	198	68.8	53.0	Manual	3D Slicer version 4.9	Fazekas
Aribisala [22]	2013	LBC 1936	Community	672	72.7	53.3	Automated	MCMxxxVI	Fazekas
Bocti [23]	2005	Unspecified	Memory clinic	75	79.5	54.3	Automated	SABRE	CHIPS
Fan [24]	2003	Unspecified	Clinical trial	220	60.9	32.3	Automated	Easy Vision 4.3	ARWMC
Gao [25]	2011	Sunnybrook Study	Memory clinic	165	70.8	49.7	Automated	ALP	Fazekas, ARWMC, Scheltens
Gouw [26]	2006	LADIS	Clinic-based	574	74	45.3	Semi-automated	In-house developed	Fazekas, Scheltens
Griffanti 1 [27]	2016	OPTIMA	Memory clinic	85	75.0	54.1	Automated	FSL	Custom VRS, ARWMC
Griffanti 2 [27]	2016	OXVASC	TIA/Stroke clinic	474	67.4	49.4	Automated	FSL	Custom VRS, ARWMC
Guerrero [28]	2018	Unspecified	Stroke clinic	167	66.0	N/R	Automated & manual	CNN (uResNet), MCMxxxVI	Fazekas DWMH, Fazekas PVWMH
Hotz [29]	2022	LHAB	Community	231	70.8	51.1	Automated	FreeSurfer v6, UBO Detector, FSL	Fazekas
Jiang [30]	2018	MAS, OATS	Community	563	72.3	45.7	Automated	FSL	Fazekas DWMH & PVWMH
Joo [31]	2022	Guro Hospital	Memory clinic	800	69.1	40	Automated	VUNO Med-DeepBrain	Fazekas DWMH & PVWMH
Kapeller [32]	2003	ASPS	Community	329	N/R	N/R	Manual	Unspecified	Fazekas, Scheltens, Manolio
Prins [33]	2004	Rotterdam Study	Community	20	72.0	50.0	Manual	Show_Images	Fazekas, Scheltens, RSS scale, Custom VRS
Schwichtenberg [34]	2017	Mannheim	Mental health institute	87	73.3	42.5	Automated	MRIcron	Fazekas, Scheltens
Sudre [35]	2018	SABRE	Community	84	71.4	61.7	Automated	In-house developed algorithm	Manolio, Fazekas, Scheltens
Tiehuis [36]	2008	Utrecht Diabetic Encephalopathy Study	Primary care & community	154	65.3	46.1	Automated	APSM	Scheltens, RSS scale
Valdés Hernández 1 [37]	2017	LBC 1921	Community	38	92.1	N/R	Automated	MCMxxxVI	Fazekas, Prins Scale
Valdés Hernández 2 [37]	2017	Stroke sample	Stroke clinic	190	66.0	58.8	Automated	MCMxxxVI	Fazekas, Prins Scale
Van den Heuvel [38]	2006	PROSPER	Community	100	74.5	59.0	Automated	software LUMC	Scheltens
Van Straaten [39]	2006	LADIS	Various settings	618	N/R	45.0	Manual	Show_Images	Fazekas, ARWMC, Scheltens
Wang [40]	2014	SNAC-K	Community	492	70.3	40.4	Manual	LST	Custom VRS
Waymont [41]	2019	1936 Aberdeen Birth Cohort	Community	243	68.0	52.0	Automated	LST	Scheltens
Wu [42]	2006	University of Pittsburgh	Centre mood disorders	19	72.2	48.1	Automated	ALP	Custom VRS, ARWMC
Xiong [43]	2011	VITATOPS	Stroke clinic	97	75.2	52.0	Automated	Easy Vision	ARWMC, Custom VRS
Zerna [44]	2018	CATCH	Stroke clinic	412	67.3	60.2	Automated	Quantomo	Total Fazekas
Zhu [45]	2022	Multi-scan IDS1, IDS2, Silver standard	General hospital	1045	62.3	53.5	Automated & manual	2D VB-Net, uResNet, 3D V-Net, VGGNet	Total Fazekas (PWMH and DWMH)

Abbreviations: n, sample size; N/R, no record; VRS, visual rating scale(s); WMH, white matter hyperintensities; PVWMH, periventricular white matter hyperintensities; DWMH, deep white matter hyperintensities; LST, Lesion segmentation tool; ALP, Automated labelling pathway.

**Fig. 3:**
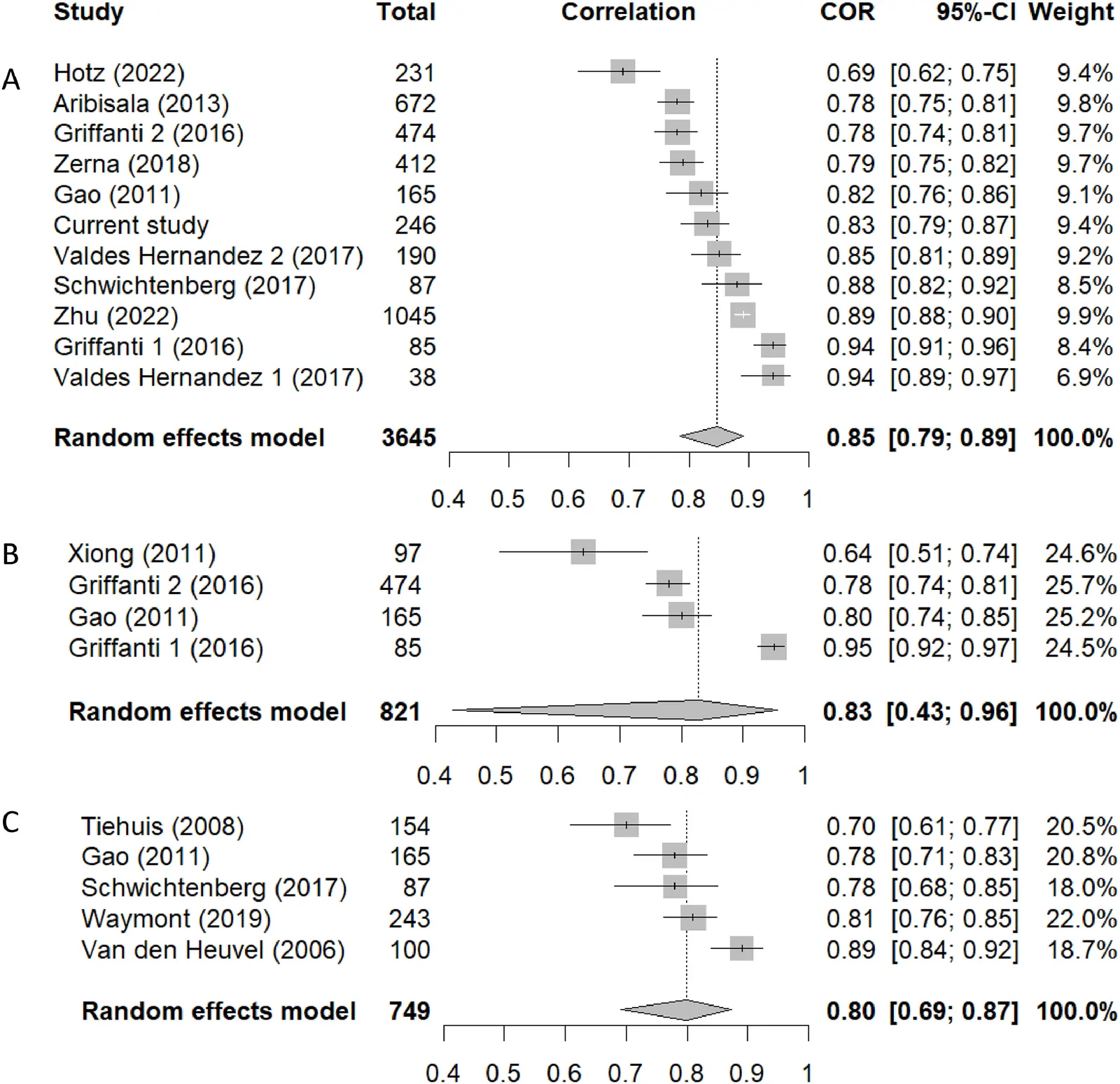
Meta-analysis of the correlation between visual rating scales and WMH segmentation.

**Subtitle:** Forest plot depicting correlations for A) Fazekas score, B) ARWMC, and C) Scheltens score. Abbreviations: COR=correlation; CI=confidence interval

In most studies VRS was assessed by multiple raters (17; 71%), though in six studies (25%) this was done by a single rater. Of the 13 studies (54%) that provided information on who performed the visual scale ratings, ten studies (42%) had ratings done by at least one neuroradiologist. Of all 24 studies, 22 (92%) did not explicitly state that raters were blinded for at least WMH volumes, while in all these studies blinding seemed plausible based on workflow or purpose of the study. The primary aim of half of the included studies (n = 12) was to validate a new VRS or segmentation tool. Further details on study quality assessment are provided in Supplementary material S2.

### Systematic review and meta-analysis: correlation between visual rating and volumetry

Of 24 studies, 13 were deemed suitable for meta-analysis as they used a VRS that was assessed in at least three studies and reported the Spearman correlation. Of the 13 studies, eight assessed the Fazekas scale, five used Scheltens’ scale, and three used the ARWMC. Across eight studies on Fazekas’ scale, volumetric data and visual rating were highly correlated (ρ=0.85, 95%CI: 0.79–0.89; Fig. 3), albeit with large heterogeneity between studies (I^2^=93%). Results were similar across the 3 studies on ARWMC (ρ=0.83; 0.44–0.95; I^2^=94%) and among 5 studies using Scheltens’ VRS (ρ=0.80; 0.69–0.87; I^2^=79%). The observed heterogeneity was not clearly attributable to the specific VRS used, as similar levels of heterogeneity were observed across studies using different scales. Excluding the Erasmus MC Alzheimer Center sample from the meta-analysis did not alter the results (ρ=0.85; 0.78–0.90; I^2^=94%).

Of the 12 studies not included in meta-analysis, five assessed the concordance between VRS and volumetry for WMH using other methods than correlation [21,24,30,32,42]. All indicated a moderate to high positive association between the methods and ANOVA showed significant differences in mean WMH volume between Fazekas score groups (Supplementary material S9) [24].

### Systematic review: comparative evidence with respect to cognition

Three studies compared visual rating and volumetry with respect to cognition. In 574 participants of the multinational Leukoaraiosis And DIsability Study (LADIS), MMSE scores were similarly correlated to the Fazekas scale, Scheltens scale, and a semi-automated volumetric method (standardised betas of −0.13, −0.13 and −0.10, respectively) [26]. A second study, embedded in the Utrecht Diabetic Encephalopathy Study, did a multidomain cognitive assessment in 102 community-dwelling patients with type 2 diabetes and 42 non-diabetic reference subjects [36]. WMH volume and Schelten’s scale were equally associated with scores on all assessed domains (standardised betas), including executive function (−0.15 vs. −0.16), processing speed (−0.22 vs. −0.25), memory (0.02 vs. −0.05), abstract reasoning (−0.17 vs. −0.10) and visuoconstruction (0.04 vs. 0.04) [36]. In a third study among 165 individuals (108 with Alzheimer’s disease, 23 mild cognitive impairment, and 34 reference subjects), a clear gradient in WMH level across cognitive groups was observed for volumetry as well as the Fazekas, ARWMC and Scheltens visual scales [25]. Volumetry, ARWMC, and Scheltens’ scale were similarly correlated with multidomain cognitive scores in the patients with Alzheimer’s disease (standardized betas ranging between −0.19 and −0.24) [25].

### Converting WMH volumes to Fazekas

One study identified in meta-analysis reported cut-off values to convert WMH volumes into Fazekas scores [31]. The extracted cut-off points to distinguish the four Fazekas scale groups were respectively at 3.4 (for Fazekas 0 vs 1, 2 or 3), 9.6 (0 or 1 vs 2 or 3) and 17.1 mL (0, 1 or 2 vs 3). When validating these cut-offs in the Erasmus MC memory clinic sample from the current study, classification accuracy of these thresholds was 59% (95% CI 50%−67%) for Fazekas 0 vs 1, 70% (59%−79%) for Fazekas 1 vs 2, and 78% (66%−87%) for Fazekas 2 vs 3, indicating lower accuracy with low volumes of WMH. When determining the volumetric cut-off points for clinically relevant distinction between Fazekas 0 vs 1, 1 vs 2, and 2 vs 3 in our sample, Youden indices pointed towards optimal cut-offs of 1.6 mL (Fazekas 0–1), 6.0 mL (Fazekas 1–2), and 19.8 mL (Fazekas 2–3), with accuracies of 74% (95%CI: 66%−81%), 84% (77%−89%) and 90% (82%−95%), respectively.

## Discussion

In this study of patients with MRI and cognitive assessment recruited from a Dutch tertiary referral centre, assessment of WMH using the Fazekas scale and automated volumetric segmentation were strongly correlated. Explained variance in cognitive performance with volumetry was marginally higher for MMSE scores, working memory, information processing speed and language tests, but not executive function. Systematic review and meta-analysis of literature findings until October 2022 confirmed the strong correlations between volumetric and visual rating measures, while highlighting also a paucity of evidence for comparing diagnostic value of the two methods in routine care.

Volumetry is commonly seen as the more refined method for expressing the amount of WMH. Despite its ordinal nature and potential interrater variability, however, our results affirm VRS as a robust tool for assessment of WMH. The systematic review did reveal considerable heterogeneity between studies, likely due to methodological and population differences. Studies applied different inclusion criteria, but also different types of VRS and rating procedures, and nearly all employed distinct segmentation software and processing pipelines, which affect volumetric estimates and complicated meta-analysis. Even within an individual study, WMH volume estimates proved sensitive to the choice of segmentation software, as illustrated by marked variation in correlations (r = 0.41 to 0.80) between VRS and volumetry with use of FreeSurfer, UBO Detector and BIANCA [29]. However, direct comparisons were limited and the use of different software and pipelines across studies precluded firm conclusions about superiority of one tool over another. Correlations may also be influenced by differences in the population characteristics, such as the distribution of WMH volume at different ages [27,37]. In spite of high correlations, volumetrics may well be more sensitive still to detect longitudinal changes in WMH volume, with potential benefits for monitoring and different types of research.

Comparing WMH appraisal methods in terms of cognitive performance in our study, volumetry did not consistently outperform visual rating. In three of five assessed domains, volumetrics performed marginally better by no >2%, whereas the opposite was observed for executive function. The team of experienced and specialised consultant neuroradiologists in our centre may have favoured the VRS, although correlation were similar in the systematic review, which included raters with various levels of experience. Two recent studies, published since the search date of our systematic review, supported similar correlation of WMH volume and Fazekas with MoCA scores, i.e. among 76 patients with hypertension (−0.47 vs. −0.57) [46], and 41 memory clinic attendees (−0.39 vs. −0.33) [47]. In contrast, WMH volume outperformed Fazekas score in terms of diagnostic value for cognitive impairment (MoCA<26) among 99 inpatients with small-vessel disease in China (AUC: 0.786 vs. 0.658) [48]. Taken together, the mostly comparable results in published literature and the modest difference in explained variance between VRS and volumetry for multiple cognitive domains in our study, challenge the assumption that more technically complex or data-intensive approaches necessarily translate into greater diagnostic or predictive value. Visual ratings may already capture much of the clinically relevant gradient of WMH severity, with limited potential gain remaining from the larger dispersion of WMH volumes within higher Fazekas categories and, on the other end, some punctiform WMH that were classified as Fazekas 0. Neither VRS nor volumetry increased explained variance by >4% beyond age, sex and education, which in itself explained maximum 17% of variance in any cognitive domain. The relatively small explained variance by a key risk indicator like age tempers expectations about the absolute increments that might be reasonably expected from WMH assessment [46]. Yet, small absolute increases in explained variance with more advanced WMH metrics may reflect substantial relative improvements (e.g., from +2 to +4% for MMSE in our study). Specific cognitive domains like processing speed may be more sensitive to WMH, even though we –unexpectedly– found no clear evidence for that in our data. Recently introduced age-specific and location-specific interpretation of WMH volume deserve further exploration [[49], [50], [51], [52], [53]]. Meanwhile, the availability and relative ease of visual rating support its use for clinical assessment of vascular cognitive impairment.

The clear distinction in WMH volume between Fazekas categories offers possibility for converting volumes into Fazekas scores for research harmonisation efforts [31]. We validated volumetric cut-offs from a prior study in our cohort. Classification performance was highest for distinguishing the upper categories, suggesting that volume-based thresholds are most reliable at higher WMH levels. In populations where overall amount of WMH is low, misclassification is to be expected particularly for the thresholds between a Fazekas score of 0 and 1. In these low ranges of WMH, visual rating could be particularly sensitive to imaging resolution and windowing, and very subtle WMH may more easily remain unclassified. Volumetric cut-offs are sensitive to differences in imaging protocols, visual rating, and volumetric segmentation. Accordingly, the proposed cut-offs from our sample warrant external validation before applying these blindly to other cohorts.

Our study is strengthened by its direct comparison between automated WMH segmentation and visual rating in a clinical sample reflective of routine care, alongside a systematic review and meta-analysis of previously published literature. Several limitations should also be considered. First, visual ratings were performed by multiple consultant neuroradiologists, which may have introduced variability, yet best reflects diagnostic value in routine practice. Similarly, differences between scanners other than field strength could have influenced results. Second, the availability of cognitive test data varied due to differences in clinical, symptom-guided diagnostic work-up. Whilst this led to variation in the sample across tests and may limit generalisability, it is unlikely to affect the internal validity of the direct comparison between Fazekas scores and automated segmentation. Still, larger studies are needed in particular to assess potential differences between cognitive domains. Third, correlation between Fazekas and segmentation was ≥0.78 in virtually all published studies, but heterogeneity was substantial and could not fully be accounted for. Due to relatively small number of studies suited for meta-analysis, meta-regression was not feasible. Fourth, blinding procedures were often insufficiently reported, and may have led to an overestimation of the estimated concordance in some studies. Fifth, reliability of automated segmentations can be affected by gliosis, and results therefore do not necessarily generalise for example to neuro-oncology outpatient clinics or post-stroke imaging. Sixth, literature was reviewed until 2022 and relevant studies published afterwards were not systematically assessed. Finally, WMH likely are of heterogeneous origin [53], and better characterisation of WMH may increase (domain-specific) explained variance in cognitive performance, in conjunction with in vivo measurements of other neurodegenerative processes that were not included in our models

In conclusion, visual and volumetric assessment provide closely aligned measures of WMH, and volumetry explained only a marginally higher share of variance in cognitive performance across specific domains among memory clinic patients. While volumetry offers opportunities for greater standardization and detailed quantification in research, the Fazekas scale remains a valid and practical tool for routine use in memory clinic settings.

## Consent statement

This study has been approved by the Medical Ethics Committee of the Erasmus MC (MEC-2016-069, MEC2023-0569, and MEC2024-0550). Written informed consent was obtained from most participants, and the local institutional review board approved for the retrospective use of anonymised data for this study.

## Funding

Dr. Wolters is supported by a Veni grant from the Netherlands Organisation for Health Research and Development (grant number 09150162010108) and a research fellowship from the Alzheimer’s Association (AARF-22-924982). The Alzheimer Center research infrastructure is further supported by Alzheimer Nederland. The funders had no role in considering the study design or in the collection, analysis, interpretation of data, writing of the report, or decision to submit the article for publication.

## CRediT authorship contribution statement

**Geke G.C.E.A. van Dijk:** Writing – original draft, Visualization, Methodology, Investigation, Formal analysis, Conceptualization. **Camiel V.J. Box:** Writing – review & editing, Supervision, Methodology, Investigation, Formal analysis, Data curation, Conceptualization. **Farog Faghir:** Validation, Methodology, Investigation, Data curation. **Joy Martens:** Writing – review & editing. **Janne M. Papma:** Writing – review & editing, Resources. **Francesco U.S. Mattace Raso:** Writing – review & editing, Resources. **Esther Van Den Berg:** Writing – review & editing, Supervision, Methodology. **Meike W. Vernooij:** Writing – review & editing, Methodology, Conceptualization. **Frank J. Wolters:** Writing – review & editing, Supervision, Resources, Methodology, Investigation, Funding acquisition, Formal analysis, Data curation, Conceptualization.

## Declaration of competing interest

None of the authors report any conflicts of interest. None of the funders had any role in the design and conduct of the study; the collection, management, analysis, and interpretation of the data; and in the preparation of the manuscript or the decision to submit for publication. Quantib provided the automated WMH segmentation software used in this study; neither Quantib nor any affiliated company had a role in the study design, data collection, analysis, interpretation, manuscript preparation, or decision to submit the manuscript for publication.
