# Supplementary material for: Visual rating versus volumetric segmentation of white matter hyperintensities for cognitive assessment: a memory clinic-based comparison and systematic review
Source: Cereb Circ Cogn Behav. 2026 Oct 3;11:100564. doi: 10.1016/j.cccb.2026.100564 (PMC13677418; doi:10.1016/j.cccb.2026.100564)
Supplement: Supplementary file 1 [file mmc1.docx]

**Supplementary Material**

**Supplementary material S1**. Literature searches systematic review

**Supplementary material S2.** Quality appraisal of studies included in systematic review

**Supplementary material S3.** Overview of cognitive test results

**Supplementary material S4.** Normality assessment

**Supplementary material S5.** Data overview of internally meta-analysed studies

**Supplementary material S6.** Sensitivity analyses for subtests of processing speed and executive functioning

**Supplementary material S7.** Variance in cognitive assessment explained by Fazekas score and WMH volume using categorical instead of continuous variables.

**Supplementary material S8.** Overview of correlation between VRS and WMH segmentation of studies not included in meta-analysis

**Supplementary material S9.** Overview of review study results using different analyses

**Supplementary material S1**. Literature searches systematic review

MedLine search:

("Algorithms"[MeSH Terms] OR "automatic segment*"[Title/Abstract] OR "automated segment*"[Title/Abstract] OR "algorithm*"[Title/Abstract] OR "volumetric*"[Title/Abstract] OR "volumetry"[Title/Abstract]) AND ("Magnetic Resonance Imaging"[MeSH Terms] OR (("magnetic resonance*"[Title/Abstract] OR "NMR"[Title/Abstract] OR "MR"[Title/Abstract]) AND ("imaging*"[Title/Abstract] OR "tomograph*"[Title/Abstract])) OR "MRI"[Title/Abstract]) AND ("Neurodegenerative Diseases"[MeSH Terms] OR "Cognitive Dysfunction"[MeSH Terms] OR "neurodegenerativ*"[Title/Abstract] OR "dement*"[Title/Abstract] OR "mild cognitive impair*"[Title/Abstract] OR "MCI"[Title/Abstract] OR "alzheimer*"[Title/Abstract] OR "parkinson*"[Title/Abstract] OR "synucleinopath*"[Title/Abstract] OR "stroke"[Title/Abstract] OR "small vessel disease"[Title/Abstract] OR "white matter hyperintensities"[Title/Abstract] OR "white matter lesions"[tiab]) AND ("Sensitivity and Specificity"[MeSH Terms] OR "pattern recognition, visual"[MeSH Terms] OR "diagnostic value*"[Title/Abstract] OR "diagnostic test*"[Title/Abstract] OR "fazekas"[Title/Abstract] OR "scheltens"[Title/Abstract] OR "GCA"[Title/Abstract] OR "diagnostic reliabilit*"[Title/Abstract] OR "diagnostic utilit*"[Title/Abstract] OR "diagnostic accurac*"[Title/Abstract] OR "predictive value*"[Title/Abstract] OR "visual rating scale*"[Title/Abstract] OR "visual assessment*"[Title/Abstract] OR "visual scale*"[Title/Abstract] OR "gold standard*"[Title/Abstract])

Embase Search:

('automated segmentation'/de OR 'segmentation algorithm'/exp OR ('volumetry'/de AND 'algorithm'/exp) OR (((automat* OR algorith*) NEAR/3 (segmentat* OR volumetr*)):ab,ti,kw) OR ((volumetric* NEAR/3 segmentation*):ab,ti,kw)) AND ('nuclear magnetic resonance imaging'/exp OR 'mri scanner'/exp OR ((('magnetic resonance*' OR nmr OR mr) NEAR/3 (imag* OR tomograph*)):ab,ti,kw) OR mri:ab,ti,kw) AND ('degenerative disease'/exp OR 'mild cognitive impairment'/exp OR neurodegenerativ*:ab,ti,kw OR dement*:ab,ti,kw OR 'mild cognitiv* impair*':ab,ti,kw OR mci:ab,ti,kw OR alzheimer*:ab,ti,kw OR parkinson*:ab,ti,kw OR synucleinopath*:ab,ti,kw OR stroke:ab,ti,kw OR 'small vessel disease':ab,ti,kw OR 'white matter hyperintensities':ab,ti,kw OR 'white matter lesions':ab,ti,kw) AND ('diagnostic value'/exp OR 'diagnostic accuracy'/de OR 'diagnostic test accuracy study'/de OR 'predictive value'/de OR 'visual rating scale'/de OR 'gold standard'/de OR 'scheltens scale'/de OR 'fazekas scale'/de OR 'fazekas score'/de OR 'prognostic value'/de OR 'prognostic assessment'/de OR ((diagnostic NEAR/3 (value* OR reliabilit* OR utilit* OR yield OR accurac*)):ab,ti,kw) OR ((predictiv* NEAR/3 value*):ab,ti,kw) OR ((visual NEAR/3 (rating OR assessment*)):ab,ti,kw) OR 'gold* standard*':ab,ti,kw OR (((scheltens OR fazekas OR gda) NEAR/3 (score* OR scale*)):ab,ti,kw) OR prognost*:ab,ti,kw) AND [english]/lim NOT ([conference abstract]/lim OR [conference review]/lim) NOT 'editorial'/de

Cochrane Search:

((((automat* OR algorith*) NEAR/3 (segmentat* OR volumetr*)) OR ((volumetric*) NEAR/3 (segmentation*))):ab,ti) AND ((((magnetic NEXT resonance* OR NMR OR MR) NEAR/3 (imag* OR tomograph*)) OR MRI):ab,ti) AND ((neurodegenerativ* OR dement* OR mild NEXT cognitiv* NEXT impair* OR MCI OR Alzheimer* OR parkinson* OR synucleinopath*):ab,ti) AND ((((diagnostic) NEAR/3 (value* OR reliabilit* OR utilit* OR yield OR accurac*)) OR ((predictiv*) NEAR/3 (value*)) OR ((visual) NEAR/3 (rating)) OR gold* NEXT standard* OR ((scheltens OR fazekas OR GDA) NEAR/3 (score* OR scale*)) OR prognost*):ab,ti) NOT "conference abstract":pt

**Supplementary material S2.** Quality appraisal of studies included in systematic review

| First author | Year | Number of raters | Function of raters | Blinding of raters to volumetrics* | Interrater reliability | Primary study aim** |
| --- | --- | --- | --- | --- | --- | --- |
| Andere^25^ | 2022 | 2 | Trained researcher, neuroradiologist | Explicit blinding | 63% | Method comparison |
| Aribisala^35^ | 2013 | 1 | Neuroradiologist | Plausible blinding (workflow) | - | Other |
| Bocti^36^ | 2005 | 2 | Unspecified | Plausible blinding (workflow) | ICC = 0.97 | VRS validation |
| Fan^21^ | 2003 | 2 | Unspecified | Plausible blinding (workflow) | К = 0.66 | VRS validation |
| Gao^37^ | 2011 | 2 | Neurologists | Plausible blinding (any clinical information, separate personnel) | Fazekas ICC = 0.88  ARWMC ICC = 0.94  Scheltens ICC = 0.89 | Clinical association |
| Griffanti^29^ | 2016 | - | Unspecified | Plausible blinding (workflow; earlier study sample with VRS) | - | Segmentation tool validation |
| Guerrero^38^ | 2018 | 1 | Radiologist | Plausible blinding (workflow) | - | Segmentation tool validation |
| Hotz^27^ | 2022 | 3 | Operators trained by neuroradiologist | Plausible blinding (workflow) | W = 0.86 | Segmentation tool validation |
| Jiang^22^ | 2018 | 2 | Neuropsychiatrist, neuroradiologist | Plausible blinding (earlier study with VRS, any clinical information) | PWMH К = 0.69  DWMH К = 0.67 | Segmentation tool validation |
| Joo^26^ | 2022 | 2 | Neuroradiologists | Plausible blinding (all clinical information) | PWMH К = 0.82  DWMH К = 0.85 | Clinical translation |
| Kapeller^23^ | 2003 | 4 | One investigator, three unspecified | One not blinded, three plausibly blinded | Baseline К = 0.59-0.78  Follow-up К = 0.09-0.39 | Method comparison |
| Prins^39^ | 2004 | 4 | Unspecified | Plausible blinding (other personnel, workflow) | Baseline PWMH ICC = 0.37  Change PWMH ICC = 0.08  Baseline DWMH ICC = 0.84  Change DWMH ICC = 0.18 | VRS validation |
| Schwichtenberg^40^ | 2017 | 2 | Unspecified | Plausible blinding (workflow) | Ratings with consensus | Other |
| Sudre^41^ | 2018 | 4 | Unspecified | Plausible blinding (goal, workflow) | Mean ICC Scheltens = 0.71  Mean ICC Manolio = 0.80  Mean ICC Fazekas = 0.64 | Other |
| Tiehuis^42^ | 2008 | 2 | Unspecified | Plausible blinding (workflow, clinical data) | ICC = 0.75-0.84 | Method comparison |
| Valdés Hernández^28^ | 2017 | 1 | Neuroradiologist | Plausible blinding (goal) | - | Other |
| Van den Heuvel^43^ | 2006 | 4 | Unspecified | Plausible blinding (workflow) | ICC Scheltens = 0.83  ICC WMH volume = 0.99 | Segmentation tool validation & comparison |
| Van Straaten^44^ | 2006 | 1 | Unspecified | Plausible blinding (clinical data, other personnel, workflow, goal) | Intra-rater К = 0.84 | Method comparison |
| Wang^45^ | 2014 | 1 | Neuroradiologist | Plausible blinding (workflow) | Intra-rater К = 0.60 | Other |
| Waymont^46^ | 2019 | >1 | Neuroradiologists | Plausible blinding (workflow) | - | Segmentation tool validation |
| Wu^24^ | 2006 | 2 | Unspecified | Plausible blinding (workflow) | - | Segmentation tool validation |
| Xiong^47^ | 2011 | 8 | 2 experienced, 6 unexperienced | Plausible blinding (workflow) | ICC = 0.85 | VRS validation |
| Zerna^48^ | 2018 | 1 | Neuroradiology fellow | Plausible blinding (workflow) | - | Other |
| Zhu^49^ | 2022 | 2 | Neuroradiologists | Explicit blinding | - | Segmentation tool validation |

Note. -, information is missing; PWMH, periventricular white matter hyperintensities measured with Fazekas; DWMH, deep white matter hyperintensities measured with Fazekas; ICC=Intraclass Correlation Coefficient; W=Kendall’s W; К =Kappa

*Blinding of raters was assessed specifically with respect to access to volumetric outcomes. Four categories were used: (i) explicit blinding to volumetric results, (ii) no blinding, (iii) plausible blinding, and (iv) unclear. Plausible blinding was assigned when blinding to volumetric results was not explicitly stated, but the reported study workflow suggested that raters were unlikely to have access to volumetric outputs (e.g. visual rating performed prior to volumetry, WMH volumetry specifically performed for purpose of the study, or application of methods by separate personnel). Plausible blinding was not considered equivalent to explicitly reported blinding.

**Primary study aim was scored in categories (i) method comparison/agreement, (ii) segmentation tool validation, (iii) VRS validation, (iv) clinical translation, (v) clinical association/prognostic relevance, and (vi) other in case the primary aim was not related to any of these categories.

**Supplementary material S3.** Overview of cognitive test results

| Cognitive domain | Test | Mean (SD) | Median (min; max) | Missing, n (%) | |
| --- | --- | --- | --- | --- | --- |
| Global cognition | MMSE | 24.2 (4.93) | 26.0 (6.00, 30.0) | | 71 (28.9) |
| Memory | Domain Z-score | 0.06 (0.98) | 0.126 (-1.91, 2.64) | | 55 (22.4) |
|  | RAVLT | 4.31 (3.30) | 4.00 (0, 13.0) | | 93 (37.8) |
|  | VAT | 8.02 (4.19) | 10.0 (0, 12.0) | | 116 (47.2) |
| Working memory | Domain score | 0.00 (1.00) | -0.127 (-2.12, 2.95) | | 109 (44.3) |
|  | WAIS-IV Digit span | 18.7 (5.52) | 18.0 (7.00, 35.0) | | 109 (44.3) |
| Language | Domain Z-score | -0.09 (0.86) | -0.151 (-2.48, 2.68) | | 19 (7.7) |
|  | BNT60 | 43.6 (12.8) | 48.0 (8.00, 60.0) | | 48 (19.5) |
|  | Animal fluency | 14.4 (6.47) | 14.0 (2.00, 37.0) | | 31 (12.6) |
|  | Letter fluency | 22.6 (13.5) | 21.0 (0, 76.0) | | 61 (24.8) |
| Processing speed | Domain Z-score | -0.04 (0.98) | 0.175 (-3.97, 1.18) | | 57 (23.2) |
|  | Stroop 1 | 66.6 (22.0) | 62.0 (38.0, 167) | | 107 (43.5) |
|  | Stroop 2 | 87.9 (30.3) | 82.0 (47.0, 253) | | 107 (43.5) |
|  | TMT-A | 80.1 (57.7) | 66.0 (12.0, 351) | | 62 (25.2) |
| Executive functioning | Domain Z-score | 0.04 (0.65) | 0.198 (-2.62, 1.01) | | 61 (24.8) |
|  | Stroop 3 | 174 (91.1) | 151 (72.0, 492) | | 106 (43.1) |
|  | TMT-B | 223 (116) | 231 (45.0, 433) | | 61 (24.8) |

**Supplementary material S4**. Normality assessment

Note. Representative Q–Q plot for analyses of TMT and Stroop illustrating the observed deviations from the diagonal at the distribution tails.

**Supplementary material S5.** Data overview of internally meta-analysed studies

| Author | Year | Sample size | Correlation coefficient | Volumetric software | Visual Rating Scale |
| --- | --- | --- | --- | --- | --- |
| Hotz | 2022 | 800 | 0.72 | FreeSurfer | Total Fazekas |
|  |  | 762 | 0.80 | UBO Detector 2D | Total Fazekas |
|  |  | 166 | 0.80 | UBO Detector 3D | Total Fazekas |
|  |  | 762 | 0.41 | BIANCA 2D | Total Fazekas |
|  |  | 166 | 0.58 | BIANCA 3D | Total Fazekas |
| Total unique scans |  | 231 | 0.69 | Combined for meta-analysis |  |
| Zhu | 2022 | 1045 | 0.89 | ITK-SNAP | Total Fazekas |
|  |  | 1045 | 0.90 | 2D VB-Net | Total Fazekas |
|  |  | 1045 | 0.89 | uResNet | Total Fazekas |
|  |  | 1045 | 0.87 | 3D V-Net | Total Fazekas |
|  |  | 1045 | 0.87 | VGGNet | Total Fazekas |
| Total |  | 1045 | 0.89 | Combined for meta-analysis |  |
| Waymont | 2019 | 243 | 0.81 | LGA | Scheltens |
|  |  | 243 | 0.82 | LPA | Scheltens |
| Total |  | 243 | 0.81 | Combined for meta-analysis |  |

Abbreviations: LGA, lesion growth algorithm; LPA, lesion probability algorithm

**Supplementary material S6.** Sensitivity analyses for subtests of processing speed and executive functioning

| Cognitive domain | Fazekas score |  | WMH volumetry segmentation |  |
| --- | --- | --- | --- | --- |
|  | Estimate (84% CI) | R^2^ | Estimate (84% CI) | R^2^ |
| Processing speed |  |  |  |  |
| TMT-A | -0.002 (-0.04; 0.03) | 0.22 | -0.05 (-0.08; -0.02) | 0.24 |
| Stroop 1 & 2 | 0.0001 (-0.0004; 0.0005) | 0.21 | 0.0005 (0.0001; 0.001) | 0.23 |
| Executive functioning |  |  |  |  |
| TMT-B | -0.08 (-0.11; -0.04) | 0.07 | -0.03 (-0.07; 0.003) | 0.04 |
| Stroop 3 | -0.02 (-0.10; 0.07) | 0.11 | -0.10 (-0.18; -0.02) | 0.13 |

Transformed, unstandardised subtest estimates were included in the models as outcome, Fazekas score and WMH volumetry segmentation as continuous determinants. All models include age, sex and educational level. Estimates reflect the change in cognitive domain composite score per unit increase in Fazekas score and 1 SD increase in WMH volume.

**Supplementary material S7.** Sensitivity analysis of explained variance of cognitive outcomes by Fazekas score and WMH volume using categorical instead of continuous variables.

|  | Fazekas score | | WMH volume | |
| --- | --- | --- | --- | --- |
| Cognitive domain | Continuous | Categorical  (score 0-3) | Continuous | Categorical (quartiles) |
| Global (MMSE)* | 0.08 | 0.09 | 0.10 | 0.08 |
| Memory | 0.15 | 0.15 | 0.15 | 0.15 |
| Working memory | 0.16 | 0.14 | 0.17 | 0.16 |
| Language | 0.18 | 0.16 | 0.19 | 0.16 |
| Processing speed | 0.10 | 0.11 | 0.12 | 0.12 |
| Executive functioning | 0.07 | 0.08 | 0.06 | 0.08 |
| All numbers represent R^2^. All models also included age, sex and educational level. *After transformation to normalise distribution. | | | | |

**Supplementary material S8.** Overview of correlation between VRS and WMH segmentation of studies not included in meta-analysis

| Author | Year | Correlation coefficient | Visual Rating Scale | Volumetric segmentation |
| --- | --- | --- | --- | --- |
| Bocti | 2005 | 0.87 | CHIPS | SABRE |
| Guerrero | 2018 | 0.77 | DWMH Fazekas | uResNet |
|  |  | 0.77 | DWMH Fazekas | DeepMedic |
|  |  | 0.75 | DWMH Fazekas | LPA |
|  |  | 0.63 | DWMH Fazekas | LGA |
|  |  | 0.77 | DWMH Fazekas | Manual delineation |
|  |  | 0.78 | PVWMH Fazekas | uResNet |
|  |  | 0.78 | PVWMH Fazekas | DeepMedic |
|  |  | 0.78 | PVWMH Fazekas | LPA |
|  |  | 0.72 | PVWMH Fazekas | LGA |
|  |  | 0.77 | PVWMH Fazekas | Manual delineation |
| Prins | 2004 | 0.5 | average of 4 VRS PVWMH | In-house developed software Show_Images |
|  |  | 0.41 | average of 4 VRS DWMH | In-house developed software Show_Images |
| Van Straaten | 2006 | 0.86 | VRS not specified | In-house developed software Show_Images |
| Wang | 2014 | 0.8 | Custom VRS | LST |

Abbreviations: PVWMH, periventricular white matter hyperintensities; DWMH, deep white matter hyperintensities; VRS, visual rating scale

**Supplementary material S9.** Overview of review study results using different analyses

| **Author** | **Year** | **Population** | **Sample size** | **VRS** | **Volumetric segmentation software** | **Analysis** | **Conclusion** |
| --- | --- | --- | --- | --- | --- | --- | --- |
| Andere | 2022 | Rhode Island Hospital | 198 | Fazekas | Manual segmentations of WMH were completed using 3D Slicer 4.9 | ANOVA to test whether WMHV differed across Fazekas scores | Fazekas scores correspond with distinct ranges of WMH volume with relatively little overlap, but scores based on volumes are more efficacious. A modified Fazekas from 0-4 should be considered. |
| Fan | 2003 | Clinical sample using MRI, Chinese University of Hong Kong | 220 | ARWMC | Easy Vision 4.3 | The correlation between volumes and rating scores was calculated with the 1-way analysis of variance (ANOVA) statistic. | The mean volume for grade 1 was 1324.59 mm3, for grade 2 was 6475.48 mm3, and for grade 3 was 25,440.05 mm3 (1-way ANOVA, P < .001). Pairwise multiple comparisons with Tamhane’s T2 showed that the differences between the means of grades 1 and 2, grades 2 and 3, and grades 1 and 3 were significant (P < .05). |
| Jiang | 2018 | OATS cohort | 397 | Fazekas | UBO detector | ANCOVA: fazekas ~ volume; and a linear mixed model: volume ~ cognition + age + sex | UBO Detector showed high agreement with manual tracing and Fazekas scores. |
| Joo | 2022 | Guro hospital | 800 | Fazekas DWMH and PVWMH; highest is considered as total Fazekas | VUNO Med-DeepBrain | The primary outcome was the diagnostic performance of automatic WMH volume and volume ratio for classifying the grades of Fazekas scale with its optimal cut-off values. Additional ANOVA among Fazekas groups. | In the training set (n = 596), optimal cut-off values of WMH volume for differentiating normal vs. mild/moderate/severe, normal/mild vs. moderate/severe, and normal/mild/moderate vs. severe were 3.4 mL, 9.6 mL and 17.1 mL, respectively.  In conclusion, the deep learning-based automatic WMH segmentation algorithm may be an accurate and promising method for classifying the grades of the Fazekas scale and differentiating subcortical vascular dementia. |
| Kapeller | 2003 | Random study sample from different European sites | 74 | Fazekas and Schmidt, Manolio, Scheltens | Philips Gyro View computer with curser-controlled manual outlining | nonparametric Kendall *W* test | There was significant agreement between all 3 visual rating scales and the quantitative volumetric measurement of ARWMC. |
| Sudre | 2018 | SABRE | 84 | Fazekas, Manolio, Scheltens | In-house developed algorithm | Kendall’s tau correlation | There was a good agreement between the various scales. In addition, visual scales and WMH volumes were strongly correlated with Kendall’s tau coefficients of 0.59 (CI = [0.53 0.62]), 0.58 (CI = [0.54 0.61]) and 0.61 (CI = [0.57 0.63]) for the Scheltens, the Manolio and the Fazekas scales respectively. |
| Wu | 2006 | University of Pittsburgh Intervention Research Center for Late-Life Mood Disorders | 19 | In-house system developed for the Cardiovascular Health Study | ALP; In-house WMH segmentation algorithm | Linear regression model | High agreement between the newly developed semi-automated approach and the visual ratings. |
